# Supplementary material for: Phosphorus-Solubilizing Bacteria Enhance Cadmium Immobilization and Gene Expression in Wheat Roots to Reduce Cadmium Uptake
Source: Plants (Basel). 2024 Jul 21;13(14):1989. doi: 10.3390/plants13141989 (PMC11280808; doi:10.3390/plants13141989)
Supplement: Supplementary file 1 [file plants-13-01989-s001.zip › plants-2998862-supplementary.pdf]

Supporting Information for

Phosphorus-solubilizing bacteria enhance cadmium immobilization and gene expression in wheat roots to reduce cadmium uptake

Delong Kan[1], Minyu Tian[1], Ying Ruan [1]*, Hui Han [2]*

a *Key Laboratory of Hunan Provincial on Crop Epigenetic Regulation and Development, College of Bioscience and Biotechnology, Hunan Agricultural University, Changsha 410128, China*

b *Collaborative Innovation Center of Water Security for the Water Source Region of the Mid-line of the South-to-North Diversion Project of Henan Province, Nanyang Normal University, Nanyang, 473061, PR China*

*Correspondence: yingruan@hotmail.com (Y.R.); 17657311626@163.com (H.H.).

Materials and methods

Transcriptome analysis of wheat roots

(1) RNA extraction

Fresh wheat roots were washed with 0.1% diethyl phosphorocyanidate (DEPC) water to remove surface dirt and then cut into small (0.5 cm long) pieces after drying on filter paper. The wheat roots were placed in a precooled RNase-free cryopreservation tube and frozen in liquid nitrogen for 0.5 hours. An MJZol Total RNA Extraction Kit was used to extract wheat root RNA. In brief, after thorough grinding, the homogenate tissues were transferred to a centrifuge tube containing 1 mL of TRIzol, shaken such that the tissue fully decomposed, and incubated at room temperature for 5 minutes. After centrifugation at 4°C for 5 minutes at 13000 × g, the upper liquid was transferred to another centrifuge tube, and 0.2 mL of precooled chloroform was added. After shaking and mixing, the mixture was incubated at room temperature for 5 minutes. After centrifugation at 4°C and 13000 × g for 15 minutes, the upper aqueous phase (400 μL) was transferred to another centrifuge tube, and an equal volume of precooled isopropanol was added, after which the mixture was incubated at room temperature for 10 minutes. After centrifugation at 4°C and 13000 × g for 10 minutes, the precipitate was dissolved in 0.1% DEPC water.

(2) Sequencing

The wheat root RNA sample was sent to Majorbio Company for sequencing. A Nanodrop 2000 instrument was used to determine the concentration and purity of the extracted RNA, agarose gel electrophoresis was used to determine the integrity of

RNA, and Agilent 5300 instrument was used to determine the RNA integrity number (RIN). The Illumina NovaSeq 6000 platform was used for sequencing.

(3) Screening and analysis of DEGs

After we obtained the read counts of genes, DEGs were identified and analyzed to investigate their functions in multiple samples (https://www.majorbio.com). DESeq2 software was used to analyze the differential expression of these genes. The screening criteria for DEGs included a false discovery rate (FDR)<0.05 and |log2(fold change (FC)|≥1. When a gene met both conditions, it was considered to be differentially expressed. DEGs were classified according to their involvement in biological processes (BPs), cellular components (CCs), and molecular functions (MFs) via the Diamond database (GO, https://github.com/bbuchfink/diamond). GOATOOLS (https://github.com/tanghaibao/GOatools) was used to perform gene ontology (GO) enrichment analysis, and KOBAS (http://kobas.cbi.pku.edu.cn/home.do) was used to conduct a kyoto encyclopedia of genes and genomes (KEGG) pathway enrichment analysis on the DEGs (Love et al., 2014). All the analyses were conducted on the Meiji Cloud platform (https://cloud.majorbio.com/).

Table S1. Quality control list of transcriptome data

Sample	Raw reads	Raw bases	Clean reads	Clean bases	Error rate(%)	Q20(%)	Q30(%)	GC content(%)
Z_M_Cd_3	70056968	1.06E+10	67433146	9.82E+09	0.0246	98.12	94.76	56.09
Z_M_Cd_2	72160074	1.09E+10	69343478	1.02E+10	0.0248	98.02	94.54	55.9
Z_M_Cd_1	66900172	1.01E+10	64204636	9.31E+09	0.0248	98.01	94.51	55.8
Z_Cd_3	70757176	1.07E+10	67926996	9.91E+09	0.0249	97.98	94.49	55.95
Z_Cd_2	79019014	1.19E+10	76169918	1.11E+10	0.025	97.94	94.34	55.6
Z_Cd_1	78870270	1.19E+10	75728310	1.1E+10	0.0247	98.07	94.65	55.69
Z_M_3	67266866	1.02E+10	64259996	9.36E+09	0.0249	98	94.46	55.22
Z_M_2	62300718	9.41E+09	59529324	8.62E+09	0.0245	98.16	94.87	54.84
Z_M_1	81879808	1.24E+10	78590834	1.14E+10	0.0248	98.04	94.58	55.13
Z_CK_3	75821894	1.14E+10	72671174	1.05E+10	0.0249	97.98	94.48	55.65
Z_CK_2	77122842	1.16E+10	73715676	1.06E+10	0.025	97.93	94.35	55.91
Z_CK_1	67177134	1.01E+10	64580556	9.38E+09	0.0247	98.06	94.67	55.99
L_M_Cd_3	60012448	9.06E+09	57461996	8.24E+09	0.0247	98.07	94.64	55.87
L_M_Cd_2	63004018	9.51E+09	60396898	8.76E+09	0.0251	97.9	94.27	55.91
L_M_Cd_1	61419030	9.27E+09	59093208	8.59E+09	0.025	97.96	94.35	55.89
L_Cd_3	67004280	1.01E+10	63936672	9.32E+09	0.0247	98.06	94.66	56.07
L_Cd_2	71871838	1.09E+10	69053294	9.95E+09	0.0249	97.99	94.47	55.6
L_Cd_1	60225704	9.09E+09	57607276	8.37E+09	0.0248	98.02	94.55	55.86

L_M_3	62821570	9.49E+09	60167718	8.78E+09	0.0256	97.72	93.77	55.15
L_M_2	64837780	9.79E+09	61864424	8.93E+09	0.0248	98.03	94.54	55.17
L_M_1	62217106	9.39E+09	59983548	8.67E+09	0.0247	98.07	94.62	55.14
L_CK_3	67786178	1.02E+10	65293456	9.41E+09	0.0251	97.91	94.25	55.7
L_CK_2	63223080	9.55E+09	60603052	8.69E+09	0.0247	98.04	94.64	56.27
L_CK_1	59644460	9.01E+09	57282512	8.31E+09	0.0246	98.08	94.71	56.13

Q20 (%) and Q30 (%): Quality evaluation is conducted on sequencing data after quality control. Q20 and Q30 refer to the percentage of bases with sequencing quality above 99% and 99.9%, respectively, in total bases. Generally, Q20 is above 85% and Q30 is above 80%.

Table S2. The up-regulated differentially expressed genes of M2-mediated Cd detoxification in wheat root

Gene_id	Gene_description
TraesCS1A02G022000	serine-type endopeptidase inhibitor activity
TraesCS1A02G041300	response to wounding
TraesCS1A02G221600	GATA transcription factor
TraesCS1A02G382200	hydrolase activity, acting on ester bonds
TraesCS1B02G234800	GATA transcription factor
TraesCS1B02G407300	hydrolase activity, acting on ester bonds
TraesCS1B02G407500	hydrolase activity, acting on ester bonds
TraesCS1D02G021900	serine-type endopeptidase inhibitor activity
TraesCS1D02G022000	Bowman-Birk trypsin inhibitor-like protein
TraesCS1D02G318700	Peroxidase
TraesCS1D02G389900	hydrolase activity, acting on ester bonds
TraesCS2A02G018500	ATP binding,Purine metabolism,hydrolase activity
TraesCS2B02G066000	metal ion binding,xidoreductase activity
TraesCS2B02G173400	Peroxidase
TraesCS2B02G310100	Function unknown
TraesCS2B02G454400	Function unknown
TraesCS2B02G613100	catalytic activity,transferase activity
TraesCS2B02G613300	oxidoreductase activity,metal ion binding
TraesCS2D02G153800	Peroxidase
TraesCS3A02G155800	water channel activity,transmembrane transport
TraesCS3A02G293700	DNA binding,transcription factor activity
TraesCS3B02G328400	DNA binding,transcription factor activity
TraesCS3B02G353700	electron carrier activity
TraesCS3B02G533800	hydrolase activity, defense response
TraesCS3D02G293500	DNA binding,transcription factor activity
TraesCS3D02G526100	Function unknown
TraesCS4A02G016600	DNA binding,sequence-specific DNA binding
TraesCS4A02G034200	Expansin
TraesCS4B02G024500	Dirigent protein
TraesCS4B02G271300	Expansin
TraesCS4B02G287600	DNA binding,transcription factor activity
TraesCS4D02G005500	iron ion binding,oxidoreductase activity
TraesCS4D02G270400	Expansin
TraesCS4D02G286400	DNA binding,transcription factor activity
TraesCS6A02G008600	Function unknown
TraesCS6A02G216500	DNA binding,nucleic acid binding
TraesCS6A02G243900	hydrolase activity, hydrolyzing O-glycosyl compounds
TraesCS6A02G290200	O-acetyltransferase activity
TraesCS6D02G011900	Function unknown

TraesCS6D02G043600	DNA binding,transcription factor activity
TraesCS7A02G412000	transferase activity, transferring glycosyl groups
TraesCS7A02G453100	GTP binding
TraesCS7B02G105200	MAPK signaling pathway - plant
TraesCS7D02G368800	oxidoreductase activity
TraesCS7D02G369700	Peroxidase
TraesCS7D02G442500	GTPase activity
TraesCS7D02G488900	Function unknown

Table S3. The up-regulated differentially expressed genes of M2-mediated Cd immobilization in wheat root

Gene ID	Gene Description
TraesCS4D02G238100	-
TraesCS7A02G201300	-
TraesCS7D02G161800	C2H2-type zinc finger protein, Abscisic acid-induced antioxidant defence, Water stress and oxidative stress toleranc [Source: Projected from Oryza sativa (Os03g0437200)]
TraesCS2A02G214400	Similar to Chitin-inducible gibberellin-responsive protein [Source: Projected from Oryza sativa (Os07g0545800)]
TraesCS5A02G477900	-
TraesCS3B02G288900	-
TraesCS2A02G104800	-
TraesCS6A02G416600	-
TraesCS3B02G045900	-
TraesCS2D02G204400	-
TraesCS3B02G310100	-
TraesCS6A02G393500	-
TraesCS1B02G399300	-
TraesCS5B02G300200	NEET [Source:Projected from Arabidopsis thaliana (AT5G51720) UniProtKB/TrEMBL;Acc:A0A178UGQ5]
TraesCS2D02G126500	-
TraesCS7B02G325400	Exocyst subunit Exo70 family protein [Source:UniProtKB/TrEMBL;Acc:A0A341YJW7]
TraesCS4A02G405400	-
TraesCS2D02G204700	Phenylalanine ammonia-lyase [Source:UniProtKB/TrEMBL;Acc:A0A341R722]
TraesCS7B02G104200	Hexosyltransferase [Source:UniProtKB/TrEMBL;Acc:A0A341YBE3]
TraesCS5B02G029300	-
TraesCS7B02G463300	-
TraesCS3B02G549500	-
TraesCS1B02G141100	-
TraesCS5B02G068600	-
TraesCS6B02G334000	-
TraesCS7D02G161200	Pathogenesis-related protein 1-4 [Source:UniProtKB/TrEMBL;Acc:Q94F73]
TraesCS7A02G509000	-
TraesCS3B02G351500	-
TraesCS4B02G194000	-
TraesCS3B02G453300	-
TraesCS3A02G471100	-

TraesCS1A02G370400	-
TraesCS7D02G490300	-
TraesCS7B02G065700	C2H2-type zinc finger protein, Abscisic acid-induced antioxidant defence, Water stress and oxidative stress toleranc [Source: Projected from Oryza sativa (Os03g0437200)]
TraesCS6D02G244000	-
TraesCS2D02G198200	Chitin-inducible gibberellin-responsive protein [Source: Projected from Oryza sativa (Os07g0583600)]
TraesCS2D02G401500	-
TraesCS6D02G295100	-
TraesCS2B02G507200	-
TraesCS2B02G401300	-
TraesCS4A02G369000	-
TraesCS5A02G198800	RING-type E3 ubiquitin transferase [Source:UniProtKB/TrEMBL;Acc:A0A341UX61]
TraesCS5A02G265100	-
TraesCS7D02G197300	-
TraesCS6D02G085100	-
TraesCS4B02G187300	-
TraesCS5D02G250300	Carboxypeptidase [Source:UniProtKB/TrEMBL;Acc:A0A1D5ZXP8]
TraesCS3B02G045500	-
TraesCS3B02G289400	-
TraesCS7D02G339700	-
TraesCS5B02G457300	-
TraesCS5B02G132500	-
TraesCS5D02G461600	-
TraesCS7D02G516100	-
TraesCS1D02G363800	-
TraesCS7A02G320800	-
TraesCS2A02G309100	-
TraesCS2B02G499700	-
TraesCS6D02G157600	-
TraesCS7B02G273400	-
TraesCS5A02G477100	-
TraesCS7B02G107900	-
TraesCS1A02G033200	-
TraesCS4B02G188800	-
TraesCS3B02G486800	-
TraesCS4A02G298400	-
TraesCS6B02G009500	-
TraesCS2A02G564800	-
TraesCS4B02G112500	-
TraesCS3D02G337400	-

TraesCS2B02G398300	-
TraesCS1A02G410500	-
TraesCS4B02G056600	-
TraesCS3A02G260100	Endochitinase [Source:UniProtKB/TrEMBL;Acc:A0A023W4F1]
TraesCS3A02G338900	-
TraesCS2D02G313400	-
TraesCS4B02G297100	-
TraesCS4A02G429000	-
TraesCS1B02G234900	-
TraesCS3B02G256500	-
TraesCS7D02G368400	-
TraesCS6B02G001000	-
TraesCS5B02G264700	-
TraesCS4A02G070700	-
TraesCS4A02G456300	-
TraesCS4A02G208900	-
TraesCS5D02G413200	Allene oxide synthase, chloroplastic [Source:Projected from Arabidopsis thaliana (AT5G42650) UniProtKB/Swiss-Prot;Acc:Q96242]
TraesCS3B02G289500	-
TraesCS4D02G055500	-
TraesCS4D02G303400	-
TraesCS6B02G259200	-
TraesCS3A02G440800	-
TraesCS3A02G406900	-
TraesCS6A02G266100	Xyloglucan endotransglucosylase/hydrolase [Source:UniProtKB/TrEMBL;Acc:A0A1D6A692]
TraesCS2D02G088900	-
TraesCS2B02G459100	Ubiquinol oxidase [Source:UniProtKB/TrEMBL;Acc:A0A341QU26]
TraesCS7D02G525000	-
TraesCS6D02G129900	-
TraesCS1A02G201400	-
TraesCS1A02G370700	-
TraesCS2B02G045100	-
TraesCS7D02G217500	Leucine-rich repeat receptor-like serine/threonine/tyrosine-protein kinase SOBIR1 [Source:Projected from Arabidopsis thaliana (AT2G31880) UniProtKB/Swiss-Prot;Acc:Q9SKB2]
TraesCS2B02G459000	Ubiquinol oxidase [Source:UniProtKB/TrEMBL;Acc:A0A341QWM9]
TraesCS6A02G256900	AP2 domain CBF protein [Source:UniProtKB/TrEMBL;Acc:K9M7D6]
TraesCS2A02G319500	-
TraesCS3A02G025400	-

TraesCS2B02G398000	Phenylalanine ammonia-lyase [Source:UniProtKB/TrEMBL;Acc:A0A1D5U587]
TraesCS7B02G483400	-
TraesCS2B02G529400	C2 domain-containing protein [Source:UniProtKB/TrEMBL;Acc:G8XUN9]
TraesCS6B02G346000	-
TraesCS2B02G096000	-
TraesCS4B02G268100	-
TraesCS4B02G228000	-
TraesCS2D02G168600	Transcription factor WRKY [Source:UniProtKB/TrEMBL;Acc:A7X9Y5]
TraesCS5D02G235600	-
TraesCS2D02G215900	-
TraesCS7D02G479400	-
TraesCS2D02G220200	Similar to Chitin-inducible gibberellin-responsive protein [Source: Projected from Oryza sativa (Os07g0545800)]
TraesCS1A02G221700	-
TraesCS6A02G224200	-
TraesCS4A02G123000	-
TraesCS2A02G031300	Exocyst subunit Exo70 family protein [Source:UniProtKB/TrEMBL;Acc:A0A1D5TJL2]
TraesCS5B02G408000	Allene oxide synthase, chloroplastic [Source:Projected from Arabidopsis thaliana (AT5G42650) UniProtKB/Swiss-Prot;Acc:Q96242]
TraesCS4A02G050000	-
TraesCS5D02G456100	-
TraesCS7B02G101700	-
TraesCS1B02G122800	Phenylalanine ammonia-lyase [Source:UniProtKB/TrEMBL;Acc:W5A9P3]
TraesCS5B02G336000	-
TraesCS7D02G497800	-
TraesCS2B02G224300	Phenylalanine ammonia-lyase [Source:UniProtKB/TrEMBL;Acc:A0A1D5UB55]
TraesCS4A02G115400	-
TraesCS4A02G007800	-
TraesCS2A02G461800	-
TraesCS2D02G307400	-
TraesCS1A02G167100	-
TraesCS3B02G374600	-
TraesCS6A02G262500	-
TraesCS2D02G104300	-
TraesCS5B02G313500	-
TraesCS3D02G483200	-

TraesCS1A02G367000	-
TraesCS3B02G288700	-
TraesCS2B02G398200	Phenylalanine ammonia-lyase [Source:UniProtKB/TrEMBL;Acc:A0A096UMG0]
TraesCS1B02G196600	Rhomboid-like protein [Source:UniProtKB/TrEMBL;Acc:W5A7B5]
TraesCS2B02G509400	-
TraesCS7A02G204300	-
TraesCS5D02G329100	-
TraesCS1A02G156100	Cytokinin riboside 5'-monophosphate phosphoribohydrolase [Source:UniProtKB/TrEMBL;Acc:A0A1D5RPF2]
TraesCS1D02G376700	-
TraesCS4D02G210300	-
TraesCS7B02G243100	-
TraesCS5A02G248100	-
TraesCS5B02G465300	-
TraesCS4B02G367000	-
TraesCS1B02G389900	-
TraesCS5D02G038800	-
TraesCS7D02G200600	Hexosyltransferase [Source:UniProtKB/TrEMBL;Acc:A0A1D6CZR3]
TraesCS7A02G176400	-
TraesCS1B02G354800	Peroxidase [Source:UniProtKB/TrEMBL;Acc:A0A341P8W9]
TraesCS7D02G180900	-
TraesCS3A02G209100	-
TraesCS4A02G415900	-
TraesCS4D02G124700	-
TraesCS3D02G238300	-
TraesCS7A02G160700	C2H2-type zinc finger protein, Abscisic acid-induced antioxidant defence, Water stress and oxidative stress toleranc [Source: Projected from Oryza sativa (Os03g0437200)]
TraesCS7D02G497300	-
TraesCS6D02G346400	Cadmium tolerance factor [Source:UniProtKB/TrEMBL;Acc:Q2MJU2]
TraesCS2D02G446300	-
TraesCS7A02G253700	-
TraesCS2A02G349300	-
TraesCS3A02G009000	-
TraesCS3A02G038300	-
TraesCS1D02G372900	-
TraesCS2B02G043200	Exocyst subunit Exo70 family protein [Source:UniProtKB/TrEMBL;Acc:A0A1D5U9G1]
TraesCS7A02G198300	Hexosyltransferase [Source:UniProtKB/TrEMBL;Acc:A0A341Y8V3]
TraesCS2A02G081400	-

TraesCSU02G001600	-
TraesCS3D02G223400	-
TraesCS3B02G052600	-
TraesCS1A02G370600	-
TraesCS7B02G081400	-
TraesCS2B02G603100	-
TraesCS5D02G318799	CBFIIIc-D3 [Source:UniProtKB/TrEMBL;Acc:A0MPJ8]
TraesCSU02G008100	-
TraesCS2A02G161500	-
TraesCS7D02G054000	-
TraesCS2B02G271700	Non-specific serine/threonine protein kinase [Source:UniProtKB/TrEMBL;Acc:W5BDD1]
TraesCS7D02G052400	CBFII-5.2 [Source:UniProtKB/TrEMBL;Acc:A0MPJ1]
TraesCS2D02G340600	-
TraesCS6A02G248400	Trehalose 6-phosphate phosphatase [Source:UniProtKB/TrEMBL;Acc:A0A1D6AA29]
TraesCS6B02G342600	Endoglucanase [Source:UniProtKB/TrEMBL;Acc:A0A1D6AT76]
TraesCS2B02G121600	-
TraesCS7B02G422600	-
TraesCS6D02G294800	-
TraesCS3D02G336400	-
TraesCS4A02G035900	-
TraesCS4D02G349300	-
TraesCS5A02G148000	Non-specific serine/threonine protein kinase [Source:UniProtKB/TrEMBL;Acc:A0A1D5YC72]
TraesCS4A02G296300	-
TraesCS2D02G138400	-
TraesCS7D02G417000	Exocyst subunit Exo70 family protein [Source:UniProtKB/TrEMBL;Acc:A0A1D6CNS2]
TraesCS4B02G311900	-
TraesCS2A02G161000	Trehalose 6-phosphate phosphatase [Source:UniProtKB/TrEMBL;Acc:A0A1D5TQB7]
TraesCS3B02G288100	-
TraesCS5B02G299000	-
TraesCS7D02G204300	-
TraesCS5B02G045100	Protein WIR1A [Source:UniProtKB/Swiss-Prot;Acc:Q01482]
TraesCS5B02G138500	-
TraesCS2A02G251800	Non-specific serine/threonine protein kinase [Source:UniProtKB/TrEMBL;Acc:V9LSK7]
TraesCS2D02G425600	Methionine aminopeptidase [Source:UniProtKB/TrEMBL;Acc:A0A1D5USK2]
TraesCS4B02G228700	RING-type E3 ubiquitin transferase [Source:UniProtKB/TrEMBL;Acc:A0A1D5XKU6]

TraesCS1A02G094900	Phenylalanine ammonia-lyase [Source:UniProtKB/TrEMBL;Acc:A0A1D5S372]
TraesCS1D02G385400	-
TraesCS7A02G174700	-
TraesCS4B02G268700	-
TraesCS2B02G293700	Exocyst subunit Exo70 family protein [Source:UniProtKB/TrEMBL;Acc:A0A341QPT2]
TraesCS7A02G508800	-
TraesCS5B02G405500	-
TraesCS7B02G110900	-
TraesCS2D02G018200	-
TraesCS5B02G312800	CBFIIIc-B10 [Source:UniProtKB/TrEMBL;Acc:A0MPJ9]
TraesCS2D02G596400	-
TraesCS2B02G106400	-
TraesCS3D02G413700	-
TraesCS7D02G068500	-
TraesCS1B02G389700	-
TraesCS7B02G107800	-
TraesCS2B02G239400	Similar to Chitin-inducible gibberellin-responsive protein [Source: Projected from Oryza sativa (Os07g0545800)]
TraesCS2B02G422500	-
TraesCS5B02G304900	-
TraesCS6A02G209000	-
TraesCS5D02G491600	-
TraesCS6B02G257000	Myb33 [Source:UniProtKB/TrEMBL;Acc:A0A182BBB2]
TraesCS7D02G174500	-
TraesCS2A02G020700	-
TraesCS5B02G552700	-
TraesCS7A02G201400	-
TraesCS5B02G209200	-
TraesCS1B02G119600	-
TraesCS5A02G337000	-
TraesCS2D02G157800	-
TraesCS5B02G442600	-
TraesCS5B02G312900	-
TraesCS4D02G210900	-
TraesCS2D02G347700	-
TraesCS5A02G403200	Allene oxide synthase, chloroplastic [Source:Projected from Arabidopsis thaliana (AT5G42650) UniProtKB/Swiss-Prot;Acc:Q96242]
TraesCS1A02G072100	Endoglucanase [Source:UniProtKB/TrEMBL;Acc:A0A1D5S0J4]
TraesCS2A02G570600	-
TraesCS2A02G389100	Receptor-like cytoplasmic kinase, Salt tolerance, Oxidative stress

	toleranc [Source: Projected from Oryza sativa (Os04g0540900)]
TraesCS4D02G021300	Hexosyltransferase [Source:UniProtKB/TrEMBL;Acc:W5EPF4]
TraesCS5D02G245300	-
TraesCS4B02G064900	-
TraesCS7B02G105600	RING-type E3 ubiquitin transferase [Source:UniProtKB/TrEMBL;Acc:A0A341YKH2]
TraesCS3A02G347500	-
TraesCS4B02G194900	-
TraesCS7D02G068100	-
TraesCS3A02G255600	-
TraesCS1D02G034600	-
TraesCS4D02G013400	-
TraesCS6A02G351100	-
TraesCS5B02G442700	-
TraesCS7D02G068200	-
TraesCS6D02G230500	Trehalose 6-phosphate phosphatase [Source:UniProtKB/TrEMBL;Acc:W5GXR8]
TraesCS7A02G215700	-
TraesCS2A02G104600	-
TraesCS7A02G203900	-
TraesCS5B02G026300	-
TraesCS2D02G211000	-
TraesCS2A02G271900	-
TraesCS5D02G406600	-
TraesCS7B02G396600	-
TraesCS3B02G474900	GT47_2B [Source:UniProtKB/TrEMBL;Acc:M5EEY5]
TraesCSU02G001500	-
TraesCS6B02G268100	AP2 domain CBF protein [Source:UniProtKB/TrEMBL;Acc:K9M7V3]
TraesCS3B02G379200	-
TraesCS5A02G383900	Glycosyltransferase [Source:UniProtKB/TrEMBL;Acc:A0A1D5YBU7]
TraesCS1B02G385300	-
TraesCS1A02G387600	-
TraesCS2A02G541100	Exocyst subunit Exo70 family protein [Source:UniProtKB/TrEMBL;Acc:A0A341QAP4]
TraesCS7A02G028300	-
TraesCS2A02G057500	-
TraesCS1D02G418000	-
TraesCS4D02G313300	-
TraesCS5B02G236900	-
TraesCS2B02G298200	-
TraesCS3B02G032400	-

TraesCS4D02G022900	-
TraesCS2D02G079200	-
TraesCS3A02G360300	-
TraesCS6A02G242700	-
TraesCS6A02G312400	Endoglucanase [Source:UniProtKB/TrEMBL;Acc:A0A1D6ABE6]
TraesCSU02G008300	-
TraesCS2D02G197600	Probable serine/threonine-protein kinase WNK11 [Source:Projected from Arabidopsis thaliana (AT5G55560) UniProtKB/Swiss-Prot;Acc:Q6ICW6]
TraesCS1A02G331800	-
TraesCSU02G069500	-
TraesCS1D02G101100	-
TraesCS6B02G396800	-
TraesCS2D02G587900	rRNA N-glycosidase [Source:UniProtKB/TrEMBL;Acc:A0A341RN37]
TraesCS3D02G433400	GT47_2D [Source:UniProtKB/TrEMBL;Acc:A0A1D5WIA7]
TraesCS7D02G204400	-
TraesCS4B02G210100	Serine/threonine protein phosphatase 2C 30 [Source:UniProtKB/TrEMBL;Acc:A0A142DDU5]
TraesCS2A02G193000	-
TraesCS3D02G484900	-
TraesCS1D02G039300	-
TraesCS5D02G242400	Hexosyltransferase [Source:UniProtKB/TrEMBL;Acc:A0A1D5ZKV8]
TraesCS1D02G106700	-
TraesCS1D02G073100	-
TraesCS1B02G250900	-
TraesCS1B02G440800	-
TraesCS3D02G272200	-
TraesCS1D02G039400	Phenylalanine ammonia-lyase [Source:UniProtKB/TrEMBL;Acc:A0A341PEA6]
TraesCS2A02G539100	-
TraesCS6A02G080400	-
TraesCS7B02G419400	-
TraesCS2A02G475600	RING-type E3 ubiquitin transferase [Source:UniProtKB/TrEMBL;Acc:A0A1D5TCE9]
TraesCS1B02G411100	-
TraesCS1A02G215500	Beta-amylase [Source:UniProtKB/TrEMBL;Acc:A0A1D5RW46]
TraesCS5B02G404000	-
TraesCS7A02G267000	-
TraesCS2B02G115300	-
TraesCS2B02G326200	-
TraesCS5B02G491100	-

TraesCS4A02G219500	-
TraesCS3A02G342600	-
TraesCS7D02G357500	-
TraesCS1D02G372400	-
TraesCS5D02G307000	-
TraesCS7D02G161700	-
TraesCS4D02G094200	-
TraesCS1D02G418700	-
TraesCS2B02G040500	Glycosyltransferase [Source:UniProtKB/TrEMBL;Acc:A0A1D5U8K8]
TraesCS6D02G113500	-
TraesCS2A02G438200	Ubiquinol oxidase [Source:UniProtKB/TrEMBL;Acc:A0A1D5T8G7]
TraesCS5A02G232400	-
TraesCS5A02G238400	-
TraesCS7B02G108400	-
TraesCS1B02G268000	-
TraesCS7A02G196000	-
TraesCS5B02G384500	-
TraesCS7A02G176700	-
TraesCS3B02G392200	-
TraesCS1B02G041400	-
TraesCS4B02G065000	-
TraesCS7A02G558500	-
TraesCS5D02G140600	-
TraesCS5B02G310300	-
TraesCS1B02G385100	-
TraesCS6A02G305800	-
TraesCS2B02G474600	-
TraesCS7D02G497700	-
TraesCS3D02G041500	-
TraesCS7D02G207000	-
TraesCS7D02G201600	RING-type E3 ubiquitin transferase [Source:UniProtKB/TrEMBL;Acc:A0A341Z7F5]
TraesCS3D02G096700	-
TraesCS7A02G394600	-
TraesCS5D02G006800	-
TraesCS1D02G376500	-
TraesCS5B02G181500	Pathogenesis-related protein 1-1 [Source:UniProtKB/TrEMBL;Acc:C3UZE5]
TraesCS7B02G092300	-
TraesCS4A02G117100	-
TraesCS4B02G311800	Exocyst subunit Exo70 family protein [Source:UniProtKB/TrEMBL;Acc:A0A1D5XFZ3]

TraesCS6A02G353200	-
TraesCS3D02G495200	-
TraesCS5D02G217400	-
TraesCS7A02G371600	-
TraesCS2A02G196400	Phenylalanine ammonia-lyase [Source:UniProtKB/TrEMBL;Acc:A0A1D5TL36]
TraesCS7B02G460000	-
TraesCS4D02G056900	-
TraesCS2B02G104400	Serine/threonine-protein kinase [Source:UniProtKB/TrEMBL;Acc:A0A1D5UEV3]
TraesCS2D02G090200	-
TraesCS5D02G188600	-
TraesCS7D02G217400	-
TraesCS3A02G299400	-
TraesCS5A02G478100	-
TraesCS5A02G439700	Pathogenesis-related protein 1-9 [Source:UniProtKB/TrEMBL;Acc:A0A0A7ACH5]
TraesCS7D02G204200	-
TraesCS3A02G255700	-
TraesCS2A02G591200	-
TraesCS1A02G290400	-
TraesCS5D02G293500	Purple acid phosphatase [Source:UniProtKB/TrEMBL;Acc:A0A341W3R0]
TraesCS1D02G148900	3-ketoacyl-CoA synthase [Source:UniProtKB/TrEMBL;Acc:A0A1D5SQ27]
TraesCS5D02G188500	-
TraesCS5B02G096300	-
TraesCS3D02G040800	-
TraesCS6B02G292100	-
TraesCS3A02G375400	-
TraesCS3B02G525200	-
TraesCS2D02G296700	RING-type E3 ubiquitin transferase [Source:UniProtKB/TrEMBL;Acc:A0A1D5UL17]
TraesCS4A02G257900	-
TraesCS6D02G083500	-
TraesCS6D02G242100	-
TraesCS3A02G209800	-
TraesCS7A02G173100	-
TraesCS7A02G195800	-
TraesCS7B02G254900	3-ketoacyl-CoA synthase [Source:UniProtKB/TrEMBL;Acc:A0A341YFB2]
TraesCS7A02G339600	Peroxidase [Source:UniProtKB/TrEMBL;Acc:A0A1D6BK39]
TraesCS2A02G190800	-

TraesCS5B02G193100	Trehalose 6-phosphate phosphatase [Source:UniProtKB/TrEMBL;Acc:A0A1D5ZBE4]
TraesCS5D02G185100	-
TraesCSU02G241600	-
TraesCS3D02G253500	-
TraesCS1A02G367200	-
TraesCS4A02G094300	-
TraesCS4A02G110100	-
TraesCS5D02G152300	-
TraesCS2B02G337700	-
TraesCS5B02G366100	CASP-like protein [Source:UniProtKB/TrEMBL;Acc:A0A1D5Z4J4]
TraesCS4A02G296000	Globulin-3A [Source:UniProtKB/TrEMBL;Acc:I6QQ39]
TraesCS5B02G138400	-
TraesCS3A02G343900	-
TraesCS7B02G108000	-
TraesCS1B02G385200	-
TraesCS5D02G478200	-
TraesCS7D02G161100	-
TraesCS3A02G227800	-
TraesCS3B02G112000	-
TraesCS6B02G020600	-
TraesCS7A02G502900	-
TraesCS2D02G597200	-
TraesCS7D02G207300	-
TraesCS7B02G453400	-
TraesCS7A02G060800	-
TraesCS2B02G106600	-
TraesCS6D02G158100	-
TraesCS1B02G385000	-
TraesCS2D02G219900	-
TraesCS3D02G209200	-
TraesCS4D02G002100	-
TraesCS1D02G432800	-
TraesCS4B02G316700	-
TraesCS1A02G378500	-
TraesCS7A02G508700	-
TraesCS5D02G073600	-
TraesCS2B02G576400	-
TraesCS1B02G089100	-
TraesCS2B02G289900	BHLH27 [Source:UniProtKB/TrEMBL;Acc:W5BCK3]
TraesCS3D02G253700	-
TraesCS1B02G415100	-
TraesCS7D02G067800	-

TraesCS7B02G105300	-
TraesCS4B02G304900	-
TraesCS1A02G410700	-
TraesCS5B02G313000	CBFIIIa-6.1 [Source:UniProtKB/TrEMBL;Acc:A0MPJ3]
TraesCS5D02G488900	-
TraesCS3D02G256600	-
TraesCS4A02G405100	-
TraesCS5D02G491400	-
TraesCS3B02G145500	-
TraesCS7D02G532100	-
TraesCS6D02G210500	-
TraesCS1D02G122600	-
TraesCS6A02G125500	-
TraesCS6B02G238000	-
TraesCS6D02G157800	-
TraesCS4D02G058200	-
TraesCS4A02G199900	-
TraesCS2A02G490500	-
TraesCS3A02G483000	Beta-1,3-glucanase [Source:UniProtKB/TrEMBL;Acc:Q4JH28]
TraesCS1B02G183500	-
TraesCS3B02G257400	-
TraesCS1B02G229000	Beta-amylase [Source:UniProtKB/TrEMBL;Acc:A0A1D5S9R7]
TraesCS2A02G476000	-
TraesCS3D02G030600	-
TraesCS6A02G032400	-
TraesCS2A02G481100	-
TraesCS2A02G016700	-
TraesCS7D02G055600	-
TraesCS5B02G261100	-
TraesCS4B02G361300	-
TraesCS1B02G440700	-
TraesCS4B02G187800	-
TraesCS3A02G229900	-
TraesCS2A02G196700	-
TraesCS6D02G157900	-
TraesCS2D02G088800	-
TraesCS2A02G053300	-
TraesCS4B02G228300	-
TraesCS4B02G305400	-
TraesCS7A02G105100	-
TraesCS1A02G070600	-
TraesCS2B02G045300	-
TraesCS2A02G384100	-

TraesCS1A02G068400	-
TraesCS1D02G372800	-
TraesCS7B02G444800	-
TraesCS6B02G257100	-
TraesCS5B02G013700	-
TraesCS2A02G447200	-
TraesCS7B02G024300	-
TraesCS1D02G259300	-
TraesCSU02G241700	-
TraesCS5D02G488500	-
TraesCS2A02G583000	-
TraesCS1B02G440500	-
TraesCS5A02G381000	-
TraesCS1B02G389800	-
TraesCS6B02G245300	-
TraesCS1D02G173800	-
TraesCS4D02G190100	-
TraesCS5D02G204700	RING-type E3 ubiquitin transferase [Source:UniProtKB/TrEMBL;Acc:A0A1D5ZRL9]
TraesCS6B02G137000	-
TraesCS3D02G113200	-
TraesCS1D02G376600	-
TraesCS3A02G502000	-
TraesCS2B02G592800	-
TraesCS1D02G360500	Serine/threonine-protein kinase [Source:UniProtKB/TrEMBL;Acc:A0A1D5SS27]
TraesCS3D02G341100	-
TraesCS1D02G103500	Phenylalanine ammonia-lyase [Source:UniProtKB/TrEMBL;Acc:A0A341PQE9]
TraesCS1D02G204800	-
TraesCS4B02G092900	-
TraesCS2A02G091100	-
TraesCS3B02G375600	-
TraesCS3B02G003700	-
TraesCS4A02G216100	Lipase-like PAD4 [Source:Projected from Arabidopsis thaliana (AT3G52430) UniProtKB/Swiss-Prot;Acc:Q9S745]
TraesCS1A02G121800	-
TraesCS1D02G002700	-
TraesCS1D02G372500	-
TraesCSU02G075000	-
TraesCS5D02G253000	-
TraesCS4A02G124900	MYB transcription factor [Source:Projected from Arabidopsis thaliana (AT1G48000) UniProtKB/TrEMBL;Acc:Q94CJ3]

TraesCS2D02G044100	-
TraesCS3D02G256400	-
TraesCS6D02G037200	-
TraesCSU02G200200	-
TraesCS3D02G045500	-
TraesCS4D02G229200	-
TraesCS6D02G033100	-
TraesCS1A02G026200	-
TraesCS3B02G584700	-
TraesCS1A02G170000	-
TraesCS4D02G134400	-
TraesCS2D02G160200	-
TraesCS7B02G024500	Glutamyl-tRNA reductase [Source:UniProtKB/TrEMBL;Acc:A0A1D6CHW5]
TraesCS2D02G596300	-
TraesCS1A02G257500	-
TraesCS7D02G317600	-
TraesCS1D02G091600	-
TraesCS2D02G186100	-
TraesCS2D02G501800	-
TraesCS5B02G312700	CBFIIIc-3.1 (Fragment) [Source:UniProtKB/TrEMBL;Acc:A0MPJ6]
TraesCS2B02G467800	-
TraesCS3D02G505700	-
TraesCS4A02G095800	-
TraesCS5D02G390700	-
TraesCS3B02G239400	-
TraesCS2B02G224000	Phenylalanine ammonia-lyase [Source:UniProtKB/TrEMBL;Acc:A0A1D5UB52]
TraesCS2A02G535900	-
TraesCS1D02G372700	-
TraesCS2D02G199800	-
TraesCS5D02G395800	Dirigent protein [Source:UniProtKB/TrEMBL;Acc:W5FXL5]
TraesCS6A02G224100	-
TraesCS5D02G446900	Pathogenesis-related protein 1-16 [Source:UniProtKB/TrEMBL;Acc:W5FY21]
TraesCS3D02G040700	-
TraesCS7B02G150000	-
TraesCS4D02G227400	-
TraesCS2A02G447500	-
TraesCS4B02G220000	-
TraesCS2D02G436200	Ubiquinol oxidase [Source:UniProtKB/TrEMBL;Acc:A0A1D5UU40]
TraesCS7B02G107700	-
TraesCS6A02G224300	MYB transcription factor SM155-1

	[Source:UniProtKB/TrEMBL;Acc:W5G730]
TraesCS1B02G202400	-
TraesCS5A02G396900	-
TraesCS7A02G164100	-
TraesCS2D02G030100	-
TraesCS4B02G355900	-
TraesCS6D02G199500	Similar to Chitinase (EC 3.2.1.14) A [Source: Projected from Oryza sativa (Os02g0605900)]
TraesCS4D02G054100	-
TraesCS2A02G309500	-
TraesCS3D02G368800	-
TraesCS5A02G364200	CASP-like protein [Source:UniProtKB/TrEMBL;Acc:A0A1D5YAY9]
TraesCS7D02G461900	-
TraesCS6B02G346400	-
TraesCS2B02G467700	-
TraesCS5A02G500700	-
TraesCS5D02G386700	-
TraesCS5D02G487900	-
TraesCS5A02G129500	-
TraesCS5D02G050100	WIR1c [Source:UniProtKB/TrEMBL;Acc:I3NUT4]
TraesCS5B02G394000	-
TraesCS6B02G127100	-
TraesCS6B02G288700	-
TraesCS7D02G157300	-
TraesCS6D02G224700	-
TraesCS7A02G055500	-
TraesCS2D02G380500	-
TraesCS5D02G410700	-
TraesCS5D02G488800	O-methyltransferase 3 [Source:UniProtKB/TrEMBL;Acc:A5HB57]
TraesCS4B02G128000	-
TraesCS5A02G132400	-
TraesCS7D02G497400	-
TraesCS4D02G195600	-
TraesCS6D02G291800	Endoglucanase [Source:UniProtKB/TrEMBL;Acc:A0A1D6B739]
TraesCS5A02G311900	C-repeat binding factor 10 [Source:UniProtKB/TrEMBL;Acc:A0A1D5YFJ9]
TraesCS2D02G013100	-
TraesCS7A02G201100	-
TraesCS3A02G441000	-
TraesCS2B02G216800	-
TraesCS5A02G229000	-
TraesCS7D02G204700	-
TraesCS1D02G395600	-

TraesCS5A02G427400	Alpha/beta hydrolase fold-3 domain containing protein [Source: Projected from Oryza sativa (Os03g0790500)]
TraesCS1B02G323100	Serine/threonine-protein kinase [Source:UniProtKB/TrEMBL;Acc:A0A1D5S6I1]
TraesCS6B02G246800	Similar to Chitinase (EC 3.2.1.14) A [Source: Projected from Oryza sativa (Os02g0605900)]
TraesCS7A02G528000	-
TraesCS6D02G240300	-
TraesCS7B02G418500	-
TraesCS5D02G074900	-
TraesCS2A02G024900	-
TraesCS5A02G300800	-
TraesCS6B02G281800	-
TraesCS6D02G037300	-
TraesCS6A02G259200	-
TraesCS5D02G216800	-
TraesCS7B02G013600	-
TraesCS1B02G384900	-
TraesCS1D02G100900	-
TraesCS1A02G367100	-
TraesCS3D02G033500	Patatin [Source:UniProtKB/TrEMBL;Acc:A0A1D5WV83]
TraesCS7A02G149000	-
TraesCS1B02G048100	Phenylalanine ammonia-lyase [Source:UniProtKB/TrEMBL;Acc:A0A1D5SL05]
TraesCS4D02G194900	-
TraesCS3A02G498900	-
TraesCS7D02G204600	-
TraesCS5D02G491500	-
TraesCS5D02G373100	CASP-like protein [Source:UniProtKB/TrEMBL;Acc:A0A1D5ZSA9]
TraesCS6A02G316000	-
TraesCS4D02G303300	-
TraesCS4D02G083100	-
TraesCS1A02G188900	Rhomboid-like protein [Source:UniProtKB/TrEMBL;Acc:A0A341NWP9]
TraesCS5A02G237900	Similar to Heat stress transcription factor Spl7 (Heat shock transcription factor) (Heat shock factor RHSF10) [Source: Projected from Oryza sativa (Os09g0456800)]
TraesCS2A02G040300	Glycosyltransferases [Source:UniProtKB/TrEMBL;Acc:A0A1D5TJ09]
TraesCS3B02G357500	-
TraesCS1D02G090600	-
TraesCS1D02G207000	-
TraesCS3A02G418100	-

TraesCS3D02G033200	Patatin [Source:UniProtKB/TrEMBL;Acc:A0A1D5WV83]
TraesCSU02G008700	Ent-kaurene synthase like 1 [Source:UniProtKB/TrEMBL;Acc:G9M5S4]
TraesCS4A02G470600	-
TraesCS7A02G308000	-
TraesCS5A02G477600	-
TraesCS5A02G380900	-
TraesCS5D02G200800	Trehalose 6-phosphate phosphatase [Source:UniProtKB/TrEMBL;Acc:A0A0C4BJY4]
TraesCS6B02G367100	-
TraesCS3B02G518900	-
TraesCS2B02G180000	-
TraesCS7A02G392000	-
TraesCS5A02G385900	Dirigent protein [Source:UniProtKB/TrEMBL;Acc:A0A341V6I4]
TraesCS4D02G188600	-
TraesCS5B02G401900	-
TraesCS7A02G073000	-
TraesCS6A02G403200	-
TraesCS1D02G187500	Rhomboid-like protein [Source:UniProtKB/TrEMBL;Acc:M1GLC1]
TraesCS6A02G377500	-
TraesCS1B02G225300	-
TraesCS7D02G177700	-
TraesCS3D02G244800	-
TraesCS6D02G316600	-
TraesCS2A02G045100	E3 ubiquitin-protein ligase WAV3 [Source:Projected from Arabidopsis thaliana (AT5G49665) UniProtKB/Swiss-Prot;Acc:Q9LTA6]
TraesCS6D02G211200	-
TraesCS3D02G080200	-
TraesCS4A02G405900	-
TraesCS1D02G223400	-
TraesCS5B02G029400	-
TraesCS7B02G221700	-
TraesCS2A02G091000	-
TraesCS6A02G262200	-
TraesCS5D02G341700	-
TraesCS1A02G110000	-
TraesCS6B02G212700	-
TraesCS1D02G384400	-
TraesCS7B02G061100	-
TraesCS2B02G217500	Chitin-inducible gibberellin-responsive protein [Source: Projected from Oryza sativa (Os07g0583600)]
TraesCS4D02G110000	-

TraesCS3D02G256500	-
TraesCS1D02G122800	-
TraesCS5D02G488300	-
TraesCS6D02G192500	-
TraesCS6A02G235100	-
TraesCS3D02G524700	Wheatwin-2 [Source:UniProtKB/Swiss-Prot;Acc:O64393]
TraesCS1A02G355300	-
TraesCS2D02G204600	Protein BRICK 1 [Source:Projected from Arabidopsis thaliana (AT2G22640) UniProtKB/Swiss-Prot;Acc:Q94JY4]
TraesCS2B02G043800	Exocyst subunit Exo70 family protein [Source:UniProtKB/TrEMBL;Acc:A0A1D5UCC1]
TraesCS5D02G219600	-
TraesCS5D02G102700	-
TraesCS7B02G478800	-
TraesCS1D02G372600	-
TraesCS1A02G255400	-
TraesCS4D02G229900	RING-type E3 ubiquitin transferase [Source:UniProtKB/TrEMBL;Acc:A0A341UK14]
TraesCS6D02G158400	-
TraesCS2D02G563000	-
TraesCS2A02G570500	-
TraesCS6D02G362100	-
TraesCS5D02G137000	-
TraesCS6A02G224400	MYB33 [Source:UniProtKB/TrEMBL;Acc:G3LZX9]
TraesCS6B02G197000	-
TraesCS3A02G252900	-
TraesCS5A02G477200	-
TraesCS2D02G509800	-
TraesCS6D02G238200	AP2 domain CBF protein [Source:UniProtKB/TrEMBL;Acc:K9M7G7]
TraesCS1D02G211200	-
TraesCSU02G198000	Dirigent protein [Source:UniProtKB/TrEMBL;Acc:A0A341ZF53]
TraesCS7B02G101500	-
TraesCS5D02G318800	AP2 domain CBF protein [Source:UniProtKB/TrEMBL;Acc:K9M7N2]
TraesCS1B02G310100	RING-type E3 ubiquitin transferase [Source:UniProtKB/TrEMBL;Acc:A0A1D6D7Y3]
TraesCS3D02G225100	-
TraesCS6A02G127700	-
TraesCS3B02G284800	-
TraesCS5B02G197400	RING-type E3 ubiquitin transferase [Source:UniProtKB/TrEMBL;Acc:A0A1D6D6F6]
TraesCS1D02G363300	WRKY51 transcriptional factor

	[Source:UniProtKB/TrEMBL;Acc:A0A0M4CLZ5]
TraesCS3B02G547600	-
TraesCS1A02G150100	-
TraesCS2A02G154900	-
TraesCS2A02G189600	Chitin-inducible gibberellin-responsive protein [Source: Projected from Oryza sativa (Os07g0583600)]
TraesCS5D02G021300	-
TraesCS5D02G491200	-
TraesCS2A02G404600	-
TraesCS4A02G095900	-
TraesCS7D02G197500	-
TraesCS7A02G057700	AP2 domain CBF protein [Source:UniProtKB/TrEMBL;Acc:K9M7E0]
TraesCS5D02G490800	-
TraesCS3D02G212000	-
TraesCS5D02G488400	-
TraesCS1D02G299400	RING-type E3 ubiquitin transferase [Source:UniProtKB/TrEMBL;Acc:A0A1D5T0L0]
TraesCS6D02G211100	-
TraesCS7A02G125600	Glutamyl-tRNA reductase [Source:UniProtKB/TrEMBL;Acc:A0A1D6BWY3]
TraesCS5A02G235300	Hexosyltransferase [Source:UniProtKB/TrEMBL;Acc:A0A1D5YGB8]
TraesCSU02G059700	-
TraesCS5A02G183300	-
TraesCS7D02G354200	-
TraesCS4B02G304600	-
TraesCS5B02G468400	Phenylalanine ammonia-lyase [Source:UniProtKB/TrEMBL;Acc:A0A1D5ZD03]
TraesCS2A02G008300	-
TraesCS3A02G131200	-
TraesCS7A02G201200	-
TraesCS3A02G246700	-
TraesCS1D02G418400	-
TraesCS3D02G540500	-
TraesCS7A02G073100	-
TraesCS3B02G309900	-
TraesCS2A02G533900	-
TraesCS2D02G281700	-
TraesCS2B02G367700	-
TraesCS1A02G016100	-
TraesCS2D02G419800	-
TraesCS2B02G151000	Serine/threonine-protein kinase

	[Source:UniProtKB/TrEMBL;Acc:A0A1D5UC34]
TraesCS6B02G419500	-
TraesCS6D02G158300	-
TraesCS5D02G490900	-
TraesCS1B02G468300	-
TraesCS6A02G099000	-
TraesCS7B02G167400	-
TraesCS2A02G501300	-
TraesCS2D02G323300	-
TraesCS6B02G179000	-

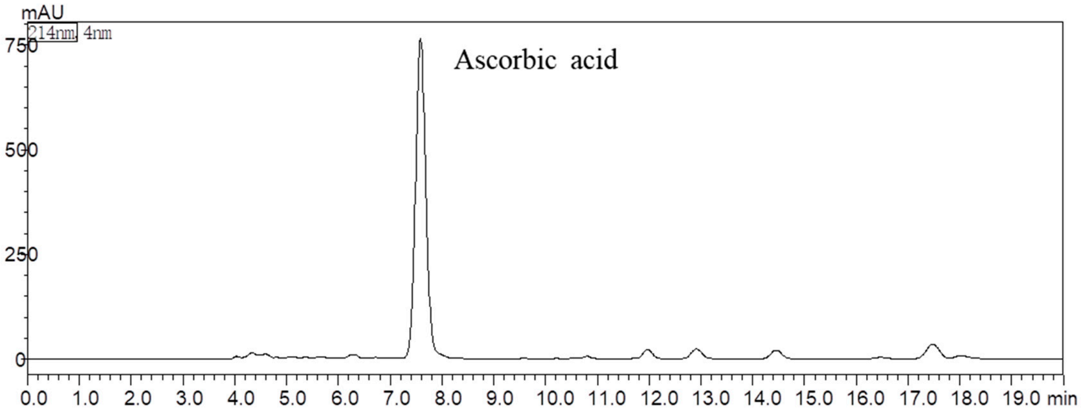

Figure S1. Determination of the ability of strain M2 to secrete organic acids under Cd stress

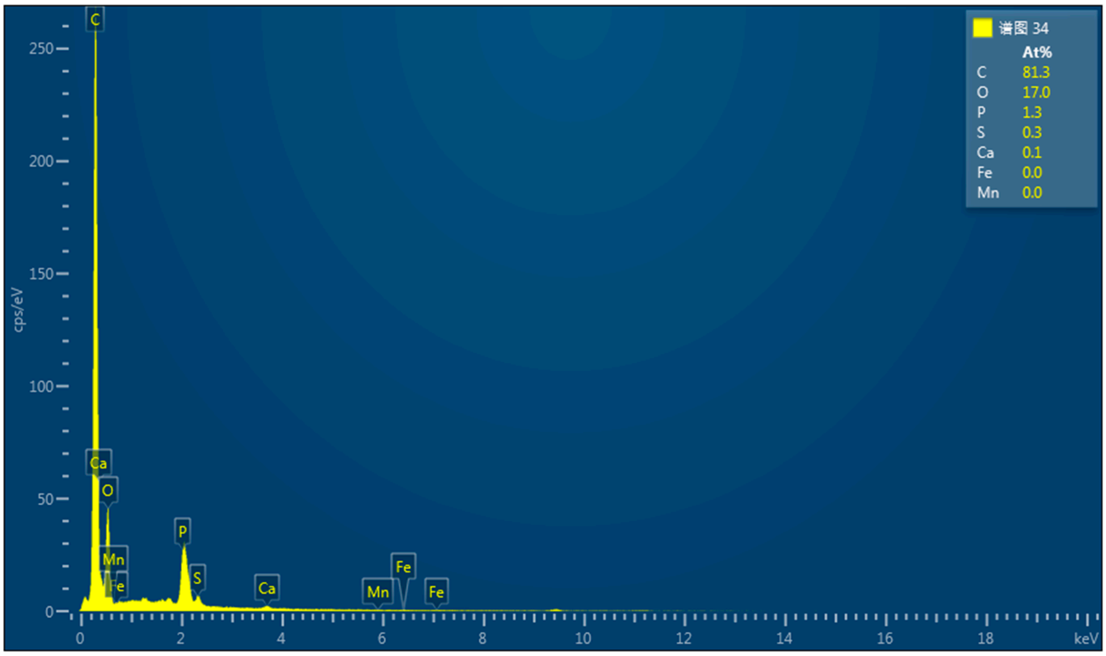

(a)

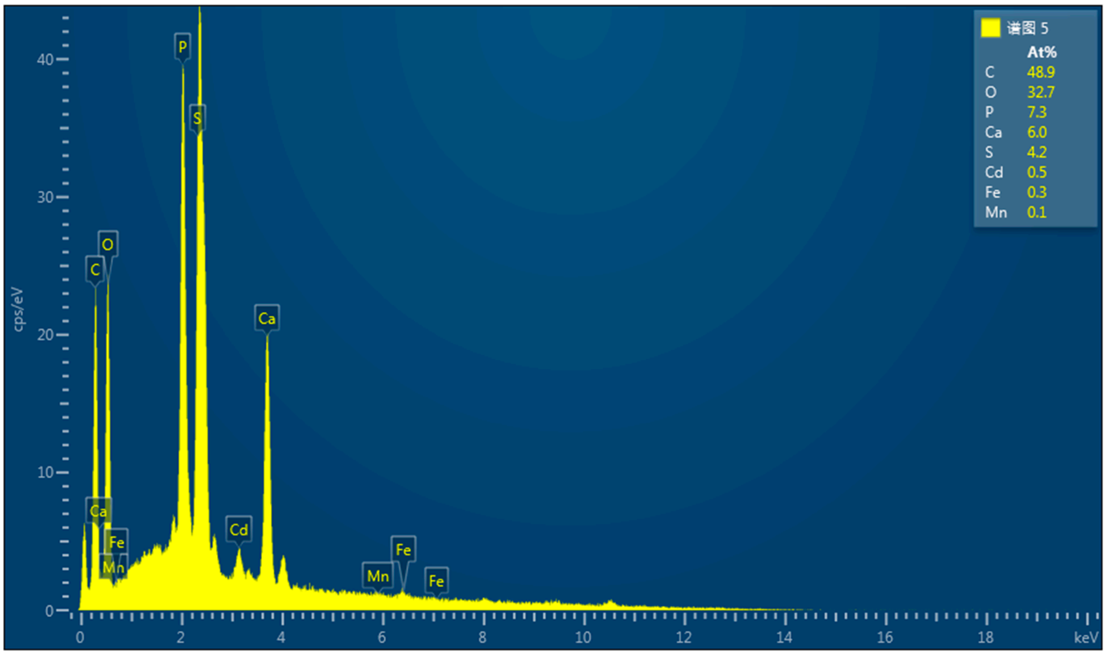

(b)

Figure S2. EDS images of strain M2 (a) and strain M2 in the presence of 5 mg L^{-1} Cd (b).

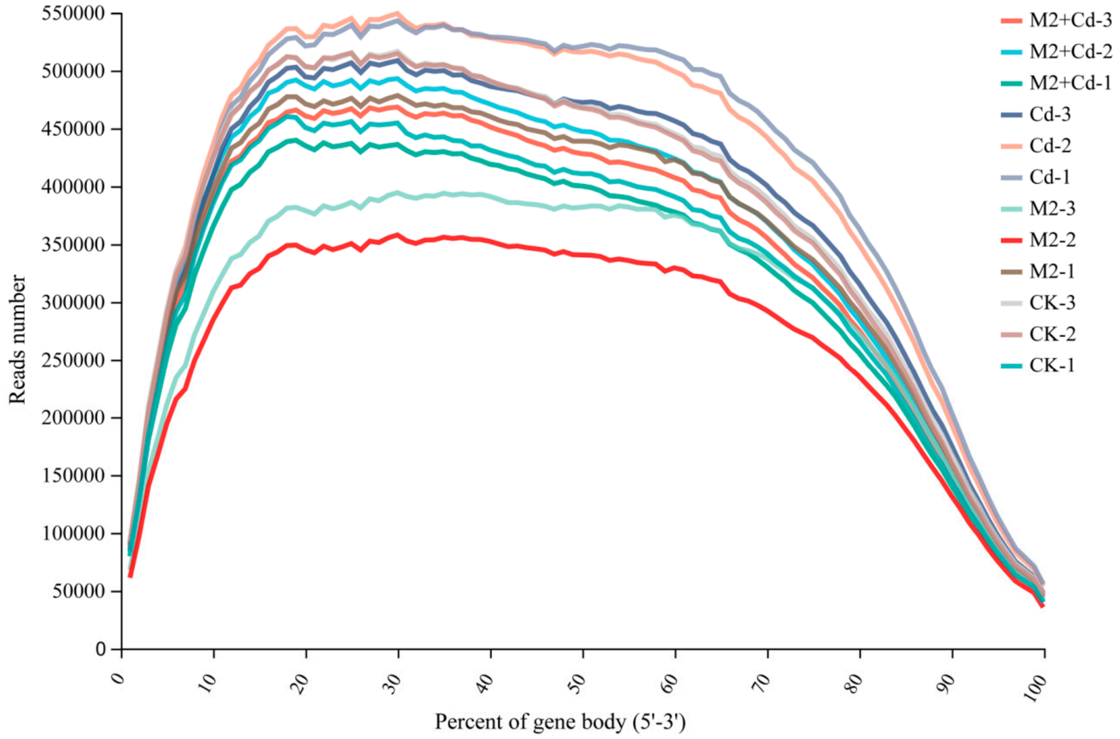

Figure S3. Sequencing coverage of transcriptome of each sample. The abscissa represents the percentage of the base length of a single gene to the total base length, with 0 representing the 5 'end of the gene and 100 representing the 3' end of the gene; The vertical axis is the sum of the number of sequences aligned to the corresponding interval on the horizontal axis of all genes.

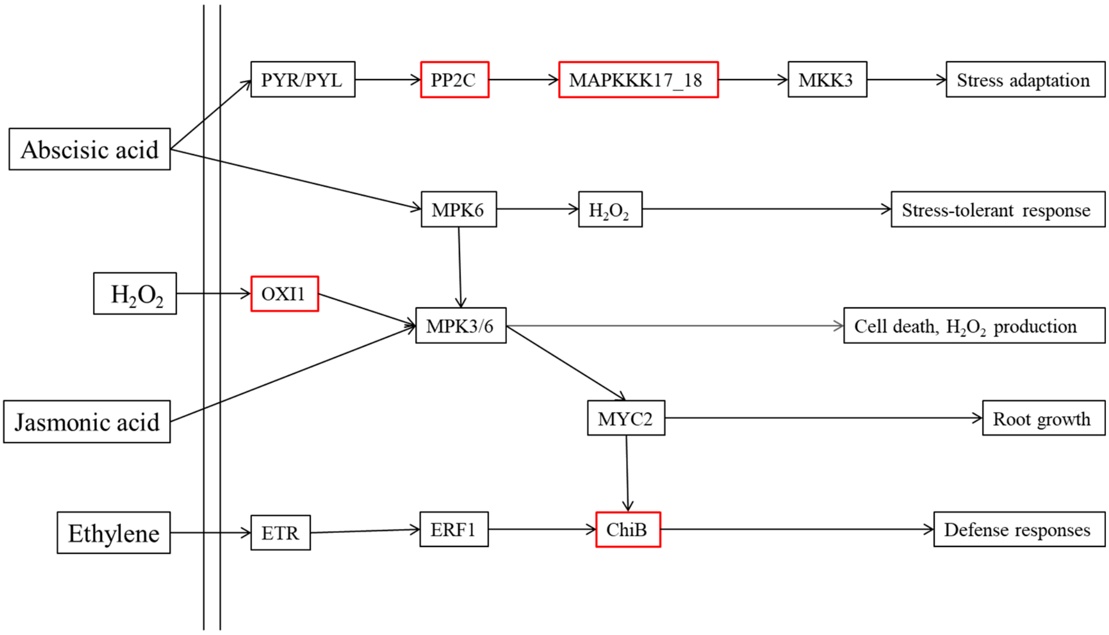

Figure S4. Key metabolic pathways in wheat roots for Cd detoxification mediated by strain M2
